# Significantly Higher Peripheral Insulin-Like Growth Factor-1 Levels in Patients With Major Depressive Disorder or Bipolar Disorder Than in Healthy Controls: A Meta-Analysis and Review Under Guideline of PRISMA

**DOI:** 10.1097/MD.0000000000002411

**Published:** 2016-01-29

**Authors:** Kun-Yu Tu, Ming-Kung Wu, Yen-Wen Chen, Pao-Yen Lin, Hung-Yu Wang, Ching-Kuan Wu, Ping-Tao Tseng

**Affiliations:** From the Department of Psychiatry, Tsyr-Huey Mental Hospital, Kaohsiung Jen-Ai's Home, Taiwan (K-YT, H-YW, C-KW, P-TT); Department of Neurology, E-Da Hospital, Kaohsiung, Taiwan (Y-WC); Department of Psychiatry, Kaohsiung Chang Gung Memorial Hospital and Chang Gung University College of Medicine, Kaohsiung, Taiwan (M-KW, P-YL); and Center for Translational Research in Biomedical Sciences, Kaohsiung Chang Gung Memorial Hospital, Kaohsiung, Taiwan (P-YL).

## Abstract

An increasing amount of research has focused on insulin-like growth factor-1 (IGF-1) because of multiple neurotrophic effects, including neurogenesis, remyelination, and synaptogenesis. In addition, IGF-1 can mediate an antidepressant effect in patients with major affective disorder, and its levels in the cerebrospinal fluid have been found to vary with antidepressant treatment. Furthermore, it has been proven to crossover the blood–brain barrier, with a reciprocal feedback loop being the central effect. However, recent studies have reported inconclusive findings about the role of IGF-1 in major affective disorder.

The aim of the current study was to conduct a thorough meta-analysis of changes in peripheral IGF-1 levels in patients with major depressive disorder (MDD) or bipolar disorder (BD). We conducted a thorough literature search and compared peripheral IGF-1 levels in patients with MDD or BD and in healthy controls, and investigated clinical variables through meta-regression.

Electronic research was conducted through platform of PubMed.

We used inclusion criteria as clinical trials discussing comparisons of peripheral IGF-1 protein levels in patients with MDD or BD and those in healthy controls.

We analyzed the cases from 9 studies with the random-effect model.

The main finding was that peripheral IGF-1 levels in the patients were significantly higher than in the healthy controls (*P* < 0.001), with a significant inverse association with duration of illness (*P* = 0.03). In meta-analysis comparing peripheral IGF-1 levels in patients with BD or MDD before and after treatment, there was no significant change in peripheral IGF-1 levels after treatment (*P* = 0.092).

The small numbers of studies and lack of correlation data with growth hormone in current studies are the main limitations of this meta-analysis.

Our results indicated that peripheral IGF-1 levels may not be an indicator of disease severity, but may be a disease trait marker or an indicator of cognition. However, further investigations on the correlation between cognitive function and peripheral IGF-1 levels are needed to explore the role of IGF-1 in the pathophysiology of MDD and BD.

## Abstract 2

Supplemental Digital Content is available in the text

## INTRODUCTION

Major affective disorders including bipolar disorder (BD) and major depressive disorder (MDD) cause patients high levels of stress and impose a substantial economic burden worldwide. Increasing evidence has shown the role of neurodegeneration and dysfunction of neurotrophic factors in the pathophysiology of BD and MDD.^1–3^ However, the current evidence still cannot fully explain the whole picture of the pathophysiology of BD and MDD.

Insulin-like growth factor-1 (IGF-1) is a member of the IGF family. It is a 70-amino acid peptide and is mainly produced in the central nervous system (CNS) and peripheral tissues such as the liver.^4,5^ IGF-1 is mainly regulated via growth hormone secretion.^6^ In addition, the secretion of IGF-1 has been shown to affect the hypothalamus-pituitary-adrenal axis reciprocally, which is believed to be one of the pathophysiologies of MDD.^7^ There are four main reasons why researchers have focused on the relationship between IGF-1 and major affective disorders. First, IGF-1 has been proven to help in neurogenesis, myelination, remyelination, neuromodulation, and synaptogenesis,^8^ which are impaired in affective disorders.^9^ In addition, it has been shown that the adult brain is more dependent on peripheral IGF-1 than the developing brain.^10^ Second, IGF-1 has been shown to exert an anti-apoptotic effect after brain damage.^11^ In addition, a previous study reported that peripheral IGF-1 mutually interacts with the CNS environment under conditions of neuroinflammation and neurodegeneration.^12^ Third, peripheral IGF-1 has been shown to mediate an antidepressant effect,^13^ and the level of IGF-1 in the cerebrospinal fluid has been shown to vary with antidepressant treatment.^14^ Finally, IGF-1 can penetrate through the blood–brain barrier (BBB),^15^ which is especially important for the development of biomarkers for MDD or BD. Both MDD and BD are thought to be diseases of the brains; however, it is difficult for clinicians to collect samples from the CNS environment in a clinical setting. Therefore, it is important to develop readily available biomarkers in peripheral tissues, such as peripheral blood, serum, or plasma.

Despite the potential role of IGF-1 in the pathophysiology of MDD and BD, there is currently a lack of conclusive evidence. In the most recent study conducted by Kopczak et al,^16^ serum IGF-1 levels were significantly higher in MDD patients than in healthy controls. Similar results have also been reported in other studies on both patients with MDD^17–19^ and BD.^20,21^ However, other studies have reported no statistically significant differences between peripheral IGF-1 levels in patients with MDD^22,23^ or BD^24^ and healthy controls. These inconclusive findings may be due to small sample sizes,^22^ different methods of detecting IGF-1,^19,24^ different sample sources,^21,24^ different times of taking the samples,^18,23^ the administration of psychotropic drugs,^16,22^ or whether or not the subjects fasted overnight.^20,23^ Therefore, in order to achieve a more conclusive result and provide a potential road of research in the biomarker or pathophysiology of major affective disorder, it should be necessary to perform a thoroughly literature review and summarization of current evidences of IGF-1 in major affective disorder.

The aims of this study were to investigate whether peripheral IGF-1 levels are different in patients with major affective disorders, including BD and MDD, compared with healthy controls, and whether this difference would vary when confounding factors varied.

## METHODS

### Literature Search and Screening

**FIGURE 1:**
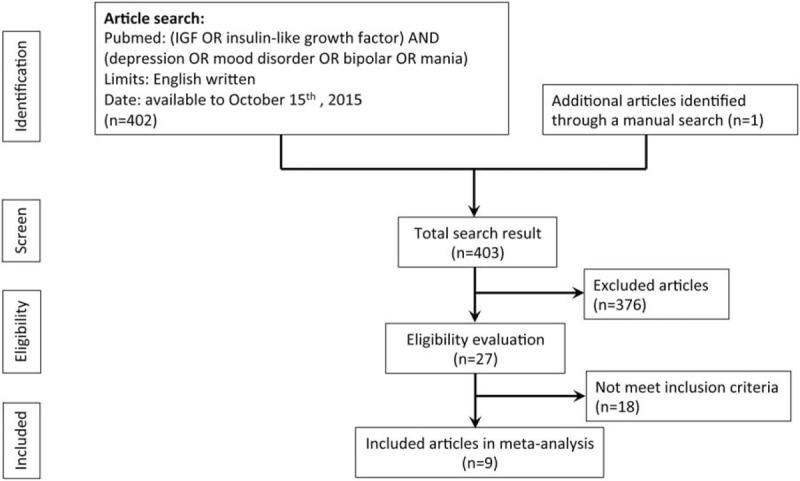
Flowchart of the selection strategy and inclusion/exclusion criteria for current meta-analysis.

The current protocol of literature research was derived from our previous report.^25^ Two independent psychiatrists (M.K. Wu and K.Y. Tu) conducted the systematic literature search using the electronic database of PubMed. If there was an inconsistent selection and lack of agreement, a final decision was made through consensus. The search was performed using the keywords “(IGF OR insulin-like growth factor) AND (depression OR mood disorder OR bipolar OR mania)” for all articles available on October 15, 2015 with the limitation of those written in English. In the first step, the search results were collected and the titles and abstracts were screened by M.K. Wu and K.Y. Tu. We excluded studies that were not related to IGF-1 in patients with BD or MDD, and thoroughly screened the remaining studies with the following inclusion criteria: articles discussing comparisons of peripheral IGF-1 protein levels in patients with MDD or BD and those in healthy controls; articles on clinical trials in humans; case-controlled trials, either in the form of preliminary reports or complete trials. The exclusion criteria were case reports or series; nonclinical trials; and those using sample sources from tissues other than peripheral blood. The screening and search protocol is shown in Figure 1. We used Jadad scores to evaluate the quality of the clinical trials in this meta-analysis.^26^

### Data Extraction

The primary outcome was peripheral levels of IGF-1 in serum, plasma, or peripheral blood. The primary outcome and other clinical variables were extracted from the included studies. When data were not available in the study, we tried to contact the authors to acquire the original data. For the peripheral levels of IGF-1, we transformed the values into a single uniform unit as far as possible in order to calculate the effect sizes (ESs).

### Meta-analytic Methods and Data Extraction

The ESs, which expressed differences in IGF-1 levels between patients with BD or MDD and healthy controls in each recruited study, were defined as the standardized mean difference based on Hedges adjusted *g*. We defined an ES greater than 0 as “higher peripheral IGF-1 levels in patients than in healthy controls.” We attempted to derive the ES from other statistical parameters such as the *t* or *P* value with the sample size when the peripheral IGF-1 levels were unavailable in the study or from the authors or when they could not be transformed into a single uniform unit. In addition, in order to evaluate the effect of treatment on peripheral IGF-1 levels, we performed meta-analysis of comparisons of the differences in peripheral IGF-1 levels in patients with BD or MDD before and after treatment. In this part of the meta-analysis, we defined an ES greater than 0 as “higher peripheral IGF-1 levels in patients after treatment than before treatment.” All of the ESs were synthesized using a random effects model for every meta-analysis.

All of meta-analysis and meta-regression procedures were performed using Comprehensive Meta-Analysis software, version 2 (Biostat, Englewood, New Jersey). We considered the analysis to be statistically significant when a 2-tailed *P* value was <0.05. Using Q statistics, related *P* values, and *I*^*2*^ statistics, we investigated the heterogeneity of each study. In addition, we investigated publication bias by visual examination of funnel plots and through Egger regression analysis.^27^ In order to investigate the possible confounding effects of clinical variables, we performed subgroup meta-analysis or meta-regression using the unrestricted maximum likelihood method. We extracted all of the clinical variables from the studies or from the original data provided by the authors, including age, gender, body mass index, duration of illness, age at onset, and disease severity according to Young Mania Rating Scale (YMRS)^28^ or Hamilton Depression rating scale (HAM-D).^29^ We defined the duration of illness as the period from the onset of first affective episode to the time when the patient entered the study. We also performed subgroup meta-analysis using patients with different disease states. Furthermore, in order to exclude the possible confounding effect of medications, we performed subgroup meta-analysis of the studies that included subjects who were drug-naive or had undergone an adequate drug wash-out period, measured the sample with enzyme-linked immunosorbent assay (ELISA) or radioimmunoassay (RIA), used sample sources from serum, plasma, or peripheral blood, used samples drawn at different times of the day, and used samples drawn from patients who did and did not fast overnight. Finally, the ethical approval was not necessary because that we did not approach the patients’ detailed data. The meta-analytic procedures used in this study fulfilled the criteria of Preferred Reporting Items for Systematic reviews and Meta-Analyses (PRISMA).^30^

## RESULTS

### Studies Included in Each Meta-Analysis

A total of 27 articles reached the screening stage, of which 4 were excluded because they focused on peripheral IGF-1 levels only in MDD patients without comparing the effects of treatment^31^ or comparisons with healthy controls,^32–34^ 4 that focused on topics not related to BD or MDD,^35–38^ 3 that were review articles,^39–41^ 4 which did not mention comparisons of peripheral IGF-1 levels,^42–45^ 1 that discussed changes in peripheral IGF-1 levels after exercise rather than clinical treatment,^46^ and 2 that were not clinical trials.^47,48^ The remaining 9 articles were then analyzed (Table 1).^16–24^ Among them, 3 included patients with BD^20,21,24^ and the other 6 included patients with MDD.^16–19,22,23^ For the quality of the clinical trials, the average Jadad score was 1.00 (Supplement Table 1).

**TABLE 1:**
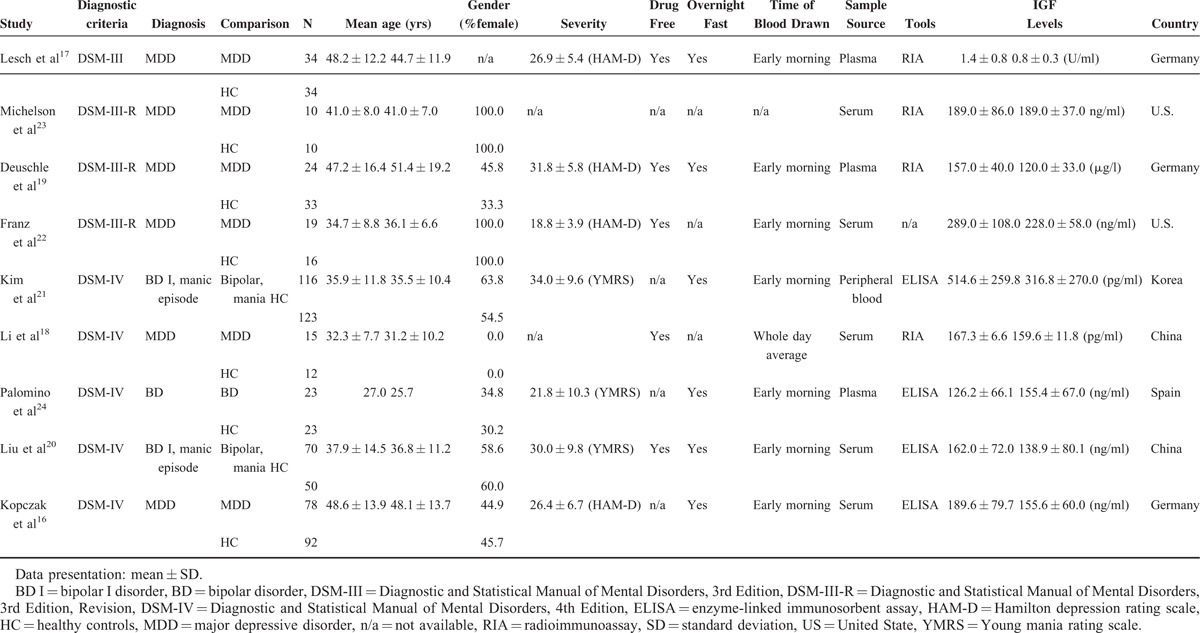
Summary of Characteristics of Studies in the Current Meta-analysis

### The Main Results of the Current Meta-analysis

**FIGURE 2:**
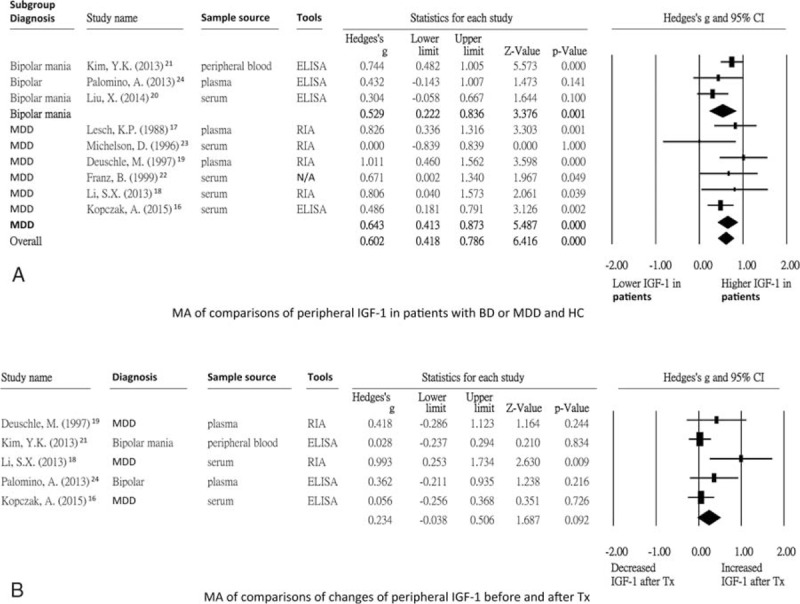
(A) Meta-analysis of comparisons of peripheral IGF-1 in patients with BD or MDD and HC; (B) Meta-analysis of comparisons of changes of peripheral IGF-1 before and after Tx. (A) indicated that the peripheral IGF-1 levels were significantly higher in patients group than HC group (*P* < 0.001). (B) indicated that there was not significant changes of peripheral IGF-1 levels after Tx (*P* = 0.092). BD = bipolar disorder, CI = confidence interval, ELISA = enzyme-linked immunosorbent assay, HC = health control, IGF-1 = insulin-like growth factor-1, MA = meta-analysis, MDD = major depressive disorder, N/A = not available, RIA = radioimmunoassay, Tx = treatment.

We included studies that compared different levels of peripheral IGF-1 in patients with BD or MDD and in healthy controls. A total of 389 patients with BD or MDD and 393 healthy controls were extracted from the 9 studies. The peripheral levels of IGF-1 in the patients with BD or MDD were significantly higher than those in the healthy controls [ESs = 0.60, 95% confidence interval (CI): 0.42–0.79, *P* < 0.001] (Figure 2A). There was no significant heterogeneity within these studies (Q = 9.82, df = 8, *I*^*2*^ = 18.51%, *P* = 0.278). The significantly higher peripheral IGF-1 levels in the patients than the healthy controls still persisted after subgroups analysis by BD and MDD (ESs = 0.53, 95% CI: 0.22–0.84, *P* = 0.001; ESs = 0.64, 95% CI: 0.41–0.87, *P* < 0.001, respectively) (Figure 2A). In addition, no significant publication bias was detected using Egger test (*t* = 0.14, df = 7, 2-tailed *P* = 0.895) and visual examination of the funnel plot. Furthermore, the results of meta-regression revealed that only the duration of illness was significantly associated with peripheral IGF-1 level (slope = −0.06, *P* = 0.03). There were no statistically significant associations between peripheral IGF-1 levels and mean age, gender (female proportion), body mass index, age at onset, YMRS score, or HAM-D score (data not shown).

Only 5 articles reported comparisons of peripheral IGF-1 levels in patients with BD or MDD before and after treatment.^16,18,19,21,24^ In the meta-analysis of these studies, we did not find any significant difference between the peripheral IGF-1 levels in patients with BD or MDD before and after treatment (ESs = 0.23, 95% CI: −0.04 to 0.51, *P* = 0.092) (Figure 2B).

### The Main Results of Subgroup Meta-analysis in Patients Who Were Drug-Naive or Had Undergone an Adequate Drug Wash-out Period

Only 5 articles included subjects who were drug-naive or had undergone an adequate drug wash-out period. Among them, 1 article included patients with BD^20^ and 4 articles included those with MDD.^17–19,22^ Therefore, we could only perform meta-analysis on the patients with MDD who were drug-naive or had undergone an adequate drug wash-out period. The results showed that the peripheral IGF-1 levels were significantly higher in the patients with MDD than in the healthy controls (ESs = 0.85, 95% CI: 0.55–1.14, *P* < 0.001).

### The Main Results of Subgroup Meta-analysis of Studies With Different Methods of Measuring Peripheral IGF-1

**FIGURE 3:**
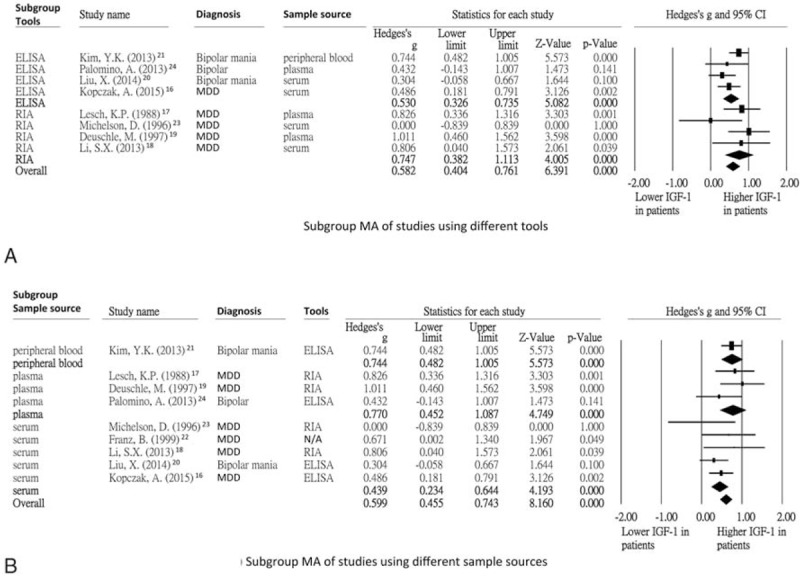
(A) Subgroup meta-analysis of studies using different tools; (B) Subgroup meta-analysis of studies using different sample sources. (A) indicated that the peripheral IGF-1 levels were both significantly higher in patients group than HC group with tools of ELISA and RIA (*P* < 0.001 and *P* < 0.001, separately). (B) indicated that the peripheral IGF-1 levels were all significantly higher in patients group than HC group with sample sources of peripheral blood, plasma, and serum (*P* < 0.001, *P* < 0.001, and *P* < 0.001, separately). BD = bipolar disorder, CI = confidence interval, ELISA = enzyme-linked immunosorbent assay, HC = health control, IGF-1 = insulin-like growth factor-1, MA = meta-analysis, MDD = major depressive disorder, N/A = not available, RIA = radioimmunoassay.

We then investigated whether different methods of measuring peripheral IGF-1 would lead to differences in the levels. Among the studies that used ELISA,^16,20,21,24^ the results showed that the peripheral IGF-1 levels were significantly higher in the patients than in the healthy controls (ES = 0.53, 95% CI: 0.33–0.74, *P* < 0.001). For the studies that used RIA,^17–19,23^ the results still showed that the peripheral IGF-1 levels were significantly higher in the patients than in the healthy controls (ES = 0.77, 95% CI: 0.46–1.08, *P* < 0.001) (Figure 3A).

### The Main Results of Subgroup Meta-analysis of Studies With Different Sample Sources

In order to investigate whether there were still significantly different peripheral IGF-1 levels after analysis by different sample sources, we performed subgroup meta-analysis with different sample sources, including peripheral blood,^21^ plasma,^17,19,24^ and serum.^16,18,20,22,23^ The results still showed significantly higher peripheral IGF-1 levels in the patients than in the healthy controls (ES = 0.74, 95% CI: 0.48–1.01, *P* < 0.001; ES = 0.77, 95% CI: 0.46–1.08, *P* < 0.001; ES = 0.44, 95% CI: 0.23–0.64, *P* < 0.001, respectively) (Figure 3B).

### The Main Results of Subgroup Meta-analysis Including Subjects Who Fasted Overnight

In a previous report, food intake was shown to affect peripheral IGF-1 levels.^49^ Therefore, in order to exclude the possible confounding effect of food intake on peripheral IGF-1 levels, we performed subgroup meta-analysis of the studies including subjects who underwent overnight fasting before blood samples were drawn.^16,17,19–21,24^ The results still revealed significantly higher peripheral IGF-1 levels in the patients than in the healthy controls (ES = 0.61, 95% CI: 0.42–0.81, *P* < 0.001).

### The Main Results of Subgroup Meta-analysis in the Early Morning

Li et al^18^ reported that peripheral IGF-1 levels vary diurnally. Therefore, we performed subgroup meta-analysis of when the blood samples were drawn. Seven used samples drawn in the early morning,^16,17,19–22,24^ 1 used samples taken throughout a “whole day,”^18^ and the other did not provide detailed information of when the blood samples were drawn.^23^ Therefore, we could only perform subgroup meta-analysis of studies using samples drawn in the early morning. The results still revealed significantly higher peripheral IGF-1 levels in the patients than in the healthy controls (ES = 0.61, 95% CI: 0.44–0.79, *P* < 0.001).

## DISCUSSION

The results of the current meta-analysis showed that peripheral IGF-1 levels were significantly higher in patients with BD or MDD than in healthy controls, in peripheral blood, plasma, or serum. Furthermore, this significance remained in subgroup meta-analysis of the use of medications, method of detecting IGF-1, overnight fasting, and different times of taking blood samples. In addition, among the clinical variables, only the duration of illness had a significantly inverse association with peripheral IGF-1 level. However, the peripheral IGF-1 level in the patients was not significantly different before and after treatment.

In a previous report, peripheral IGF-1 was proven to cross the BBB,^15^ with a reciprocal feedback loop being the central effect of IGF-1.^12,21^ This provided the rationale for clinicians to check peripheral IGF-1 levels to determine the central effect of IGF-1. Increased levels of peripheral IGF-1 indicate that the central effects of IGF-1 have decreased. The mechanism of this reciprocal interaction is controversial; however, it is believed that it may be due, at least in part, to compensatory regulation of the decreased bioavailability of IGF-1 in the CNS.^20,45,50^ It has been reported that the bioavailability of IGF-1 in the CNS environment may depend on decreased sensitivity of IGF-1 receptors under neuro-inflammatory stress.^12^

In the current meta-analysis, we found that the significantly higher peripheral IGF-1 levels in the patients than in the healthy controls were not affected by the presumed confounding factors, including whether or not the subjects were drug free, different methods of IGF-1 detection, different sample sources, different times of taking blood samples, or whether the subjects fasted overnight. These findings together with the evidence of reciprocal peripheral and central interactions suggest that the central effect of IGF-1 would be decreased in patients with BD or MDD. In fact, the decreased central effect of IGF-1, or an increased peripheral IGF-1 level, has been shown to correlate with the negative symptoms of psychotic patients. Palomino et al^24^ found a positive correlation between the scales of negative symptoms, including cognitive deficits,^51^ and peripheral IGF-1 concentration. This finding is supported by a study on another neurodegenerative disease, Huntington disease, in which patients with higher plasma IGF-1 levels were significantly associated with more severe cognitive decline.^52^ Furthermore, it is known that a decline in cognitive function occurs in patients with major affective disorders.^53^ Taken together, increased peripheral IGF-1 levels may represent a decrease in the central effect of IGF-1 and a decline in cognitive function in such patients. However, in the current meta-analysis, we could not perform further meta-regression or other analysis to prove this hypothesis because too few studies provided data on cognitive function.

An interesting result in our meta-analysis is the significant inverse association between peripheral IGF-1 levels and the duration of illness. Theoretically, a longer duration of illness should be associated with poorer cognitive function in patients with major affective disorders based on the findings discussed above, and therefore, the peripheral IGF-1 concentration would have an inverse association with cognitive function. The peripheral IGF-1 level should thus theoretically be positively associated with the duration of illness. From another point of view, the inverse association found in the current meta-analysis may be explained by somatopause, which means that the secretion of IGF-1 deceases with advancing age in healthy adults.^54,55^

In the current meta-analysis, the peripheral IGF-1 levels did not significantly vary after adequate treatment in the patients. This result is similar to previous reports in which plasma IGF-1 levels did not change after treatment with antipsychotics in animals or humans.^56,57^ In addition, there were no significant associations between the peripheral IGF-1 levels and disease severity as assessed by the YMRS and HAM-D. This indicates that peripheral IGF-1 levels may not be an indicator of disease severity, but may be a disease trait marker, or as discussed above, an indicator of cognition in such diseases.

## LIMITATIONS

There are several limitations to the current meta-analysis. First, the total number of studies included was relatively small, and this may have undermined the clinical significance. In addition, in the meta-analysis comparing peripheral IGF-1 levels before and after treatment, only 5 studies were included. Therefore, the application of our results in clinical research should be made cautiously. Second, we could not analyze an association between cognitive function and peripheral IGF-1 levels because of a lack of data. This limits the power of our results to prove the hypothesis of a relationship between peripheral IGF-1 levels and cognitive function. Finally, because peripheral IGF-1 is the downstream product of growth hormone, the concentration of peripheral IGF-1 would theoretically be affected by the concentration of growth hormone. Investigating the relationship between peripheral IGF-1 and growth hormone levels would have provided further information on the role of IGF-1 in the pathophysiology of major affective disorders. However, this could not be performed because there was a lack of such data in the studies included.

## CONCLUSION

The results of this meta-analysis showed significantly higher peripheral IGF-1 levels in the patients with MDD or BD than in healthy controls, and an inverse association with the duration of illness. Furthermore, the peripheral IGF-1 levels did not significantly vary after adequate treatment in the patients. This suggests that peripheral IGF-1 levels may not be an indicator of disease severity, but may be a disease trait marker or an indicator of cognition in such diseases. However, further investigations on the correlation between cognitive function and peripheral IGF-1 levels are needed to explore the role of IGF-1 in the pathophysiology of major affective disorders.

## Supplementary Material

Supplemental Digital Content
